# African Non-Human Primates Host Diverse Enteroviruses

**DOI:** 10.1371/journal.pone.0169067

**Published:** 2017-01-12

**Authors:** Illich Manfred Mombo, Alexander N. Lukashev, Tobias Bleicker, Sebastian Brünink, Nicolas Berthet, Gael D. Maganga, Patrick Durand, Céline Arnathau, Larson Boundenga, Barthélémy Ngoubangoye, Vanina Boué, Florian Liégeois, Benjamin Ollomo, Franck Prugnolle, Jan Felix Drexler, Christian Drosten, François Renaud, Virginie Rougeron, Eric Leroy

**Affiliations:** 1 International Center for Medical Research of Franceville, BP769, Franceville, Gabon; 2 Laboratoire MIVEGEC (Maladies Infectieuses et Vecteurs: Ecologie, Génétique, Evolution et Contrôle), Unité Mixte de Recherche (UMR), Centre National de la Recherche Scientifique (CNRS) 5290 –Institut de Recherche pour le Développement (IRD) 224 –Université de Montpellier, Montpellier, France; 3 Chumakov Institute of Poliomyelitis and Viral Encephalitides, Moscow, Russia; 4 Institute of Molecular Medicine, Sechenov First Moscow State Medical University, Moscow, Russia; 5 Institute of Virology, University of Bonn Medical Centre, Bonn, Germany; 6 CHRU de Montpellier, Montpellier, France; 7 Laboratoire Retrovirus, UMI 233, IRD University of Montpellier I, Montpellier, France; 8 German Center for Infection Research, partner Bonn—Cologne, Germany; Centers for Disease Control and Prevention, UNITED STATES

## Abstract

Enteroviruses (EVs) belong to the family *Picornaviridae* and are responsible for mild to severe diseases in mammals including humans and non-human primates (NHP). Simian EVs were first discovered in the 1950s in the Old World Monkeys and recently in wild chimpanzee, gorilla and mandrill in Cameroon. In the present study, we screened by PCR EVs in 600 fecal samples of wild apes and monkeys that were collected at four sites in Gabon. A total of 32 samples were positive for EVs (25 from mandrills, 7 from chimpanzees, none from gorillas). The phylogenetic analysis of VP1 and VP2 genes showed that EVs identified in chimpanzees were members of two human EV species, *EV-A* and *EV-B*, and those identified in mandrills were members of the human species *EV-B* and the simian species *EV-J*. The identification of two novel enterovirus types, EV-B112 in a chimpanzee and EV-B113 in a mandrill, suggests these NHPs could be potential sources of new EV types. The identification of EV-B107 and EV90 that were previously found in humans indicates cross-species transfers. Also the identification of chimpanzee-derived EV110 in a mandrill demonstrated a wide host range of this EV. Further research of EVs in NHPs would help understanding emergence of new types or variants, and evaluating the real risk of cross-species transmission for humans as well for NHPs populations.

## Introduction

Enteroviruses (EVs) are members of the genus *Enterovirus* belonging to the family *Picornaviridae* and comprise about 120 established types [1]. EVs are ubiquitous small non-enveloped RNA viruses that have a single stranded positive-sense polyadenylated genome of approximately 7.5kb flanked at 5' and 3' ends by untranslated regions (UTRs) [2]. The genome encodes a single polyprotein that is cleaved into four structural proteins (VP1 to VP4) and seven non-structural proteins (2A to 2C and 3A to 3D) [3]. In the past, the classification of EV species into serotypes was based on their antigenic and pathogenic properties in humans and laboratory animals [1]. Today, with the development of molecular typing [4], EVs are classified into nine species named *Enterovirus A* to *H* and *Enterovirus J* (*EV-A* to *EV-H* and *EV-J*). EVs infecting humans are found in the four species *EV-A* to *EV-D*. Usually the infection is asymptomatic, but enteroviruses can cause severe infections such as meningitis, encephalitis and paralytic poliomyelitis [5]. Moreover, EVs are an important source of emerging infection, such as the neuropathogenic EV-A71 [6]. The species *EV-E* and *EV-F* or bovine enteroviruses are known to infect bovines, and *EV-G* or porcine enteroviruses infect pigs [1]. The simian enteroviruses, commonly found in non-human primates (NHPs), are classified into species *EV-J* and *EV-H* [1]. Besides the species *EV-J* and *EV-H*, some EVs isolated from primates are members of the “human” species *EV-A*, *EV-B*, *EV-C* and *EV-D* [7–12], suggesting that transfers between humans and NHPs are relatively common.

EVs are cytolytic viruses and can establish persistent infections in human and animals [13, 14]. The persistence has been suggested as a major mechanism in enteroviral pathogenesis of various syndromes such as post-polio syndrome, chronic fatigue syndrome, chronic myocarditis and dilated cardiomyopathy (cited by [15]).

The simian EVs were originally isolated from captive and wild-caught primates that were commonly used in biomedical research in the 1950s and 1960s. Particularly, these EVs were isolated from Old World monkeys (OWM) of the species *Macaca mulatta* (rhesus macaque), *Macaca fascicularis* (*Cynomolgus* monkey), *Cercopithecus aethiops* (vervet monkey) and *Papio* species (baboon) [7, 10, 16, 17]. Then, other EV types were identified in fecal samples obtained from captive NHPs of the following species: *M*. *mulatta*, *M*. *nemestria* (pigtail macaque) and *Cercocebus atys* (sooty mangabey) [12, 18] presenting diarrheal disease. More recently, studies performed in Cameroon described for the first time simian EV types in wild great apes of the species *Pan troglodytes* (chimpanzee) and *Gorilla gorilla gorilla* (gorilla), and in an OWM of the species *Mandrillus sphinx* (mandrill) in Cameroon [8, 9, 19].

In the last decades, pathogens such as *Plasmodium* species [20–24], respiratory viruses [25, 26], simian immunodeficiency viruses [27], herpesvirus [28], anthrax [29] and Ebola virus [30, 31] were identified in different NHP species. Thus, pathogens of NHPs can be readily exchanged with humans, making NHPs major sources of human pathogens [32, 33]. We characterized the diversity of EVs that circulate naturally in NHPs to identify their relationship with human EV variants, the host range of enteroviruses and the capacity of primates as a source of novel EV types.

In the present study, using 600 NHPs fecal samples collected in Gabon, we analyzed the genetic diversity of EVs in two wild great apes species (*G*. *gorilla gorilla* and *P*. *troglodytes*) and in four monkey species including mandrills (*M*. *sphinx*), moustached monkey (*Cercopithecus cephus*), greater spot-nosed monkey (*Cercopithecus nictitans*) and black colobus (*Colobus satanas*). Based on phylogenetic analysis of the VP1 and VP2 genes, we studied the relationship between these simian EVs and human EVs. This work showed that simian as well as human EVs circulate naturally among chimpanzees and mandrills in Gabon, and allowed the characterization of two new *EV-B* types.

## Material and Methods

### Samples collection and study sites

**Fig 1:**
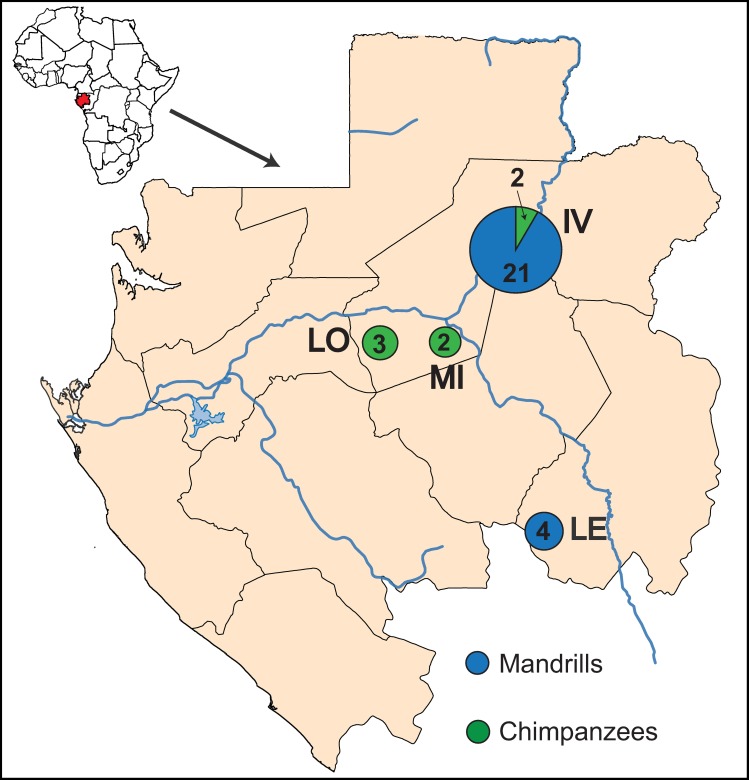
Non-human primates sampling sites in Gabon. For each site, the zone code is indicated: IV for Ivindo, LE for Lékédi, LO for Lopé, MI for Mikongo. The circles indicate positive EV samples detected in this study; numbers inside circles indicate the number of positive EV samples. Host species that yielded EVs are indicated in blue for chimpanzee and green for mandrills.

A total of 600 fecal samples of NHPs have been collected between April 2009 and October 2010 from wild-living primate communities in Gabon. Specifically, samples were obtained from four remote sites, including National Parks or forest reserves such as IV for Ivindo (S 0°09.713’; E 12°31.162’), LE for Lékédi (S 1°45.797’; E 12°57.103’), LO for Lopé (S 0°12.461’; E 11°34.043’), MI for Mikongo (S 0°16’33.915”; E 11°42’20.002”), mainly located in the central part of Gabon (Fig 1). Fresh fecal samples were collected around night nests, feeding sites and where primates were seen. Approximately 15 g of feces were gathered, preserved in RNA*later* (Ambion, Austin, TX, USA) [27], stored in the base camps at room temperature for a maximum of 15 days and then conserved at CIRMF at -80°C. In the field, the information recorded for each sample included GPS position of the site, estimation of the time of decomposition of the feces as well as morphological and/or physical aspect of the sample. The identification of the NHP species has been provisionally done in the field according to cues such as the type of nest near which the feces were found, footprints, textures and odors. In this study, fecal samples of chimpanzees (*Pan troglodytes troglodytes*), gorillas (*Gorilla gorilla gorilla*), mandrills (*Mandrillus sphinx*) and of three other monkey species such as moustached monkey (*Cercopithecus cephus*), greater spot-nosed monkey (*Cercopithecus nictitans*) and black colobus (*Colobus satanas*) have been collected.

### Research approval

Research approval for the use of NHP fecal samples was obtained from the National Center of Scientific and Technologic Research (CENAREST), permission number AR0031/09.

### Ribonucleic acid extraction and enterovirus amplification

RNA was extracted from fecal samples using EZ1 Virus Mini kit (Qiagen, Hilden, DE) according to the manufacturer’s recommended procedure. Extracted RNA were mixed in pools of five samples from the same host species. Enteroviruses screening was performed by amplifying the 5’-untranslated region (5’-UTR) through a one-step real-time reverse-transcription PCR [34]. The 95% detection limit of this assay was reported to be 100 RNA copies per reaction, which corresponds to 2.46x10e4 RNA copies per gram of feces in our settings. Individual samples of each positive pool were screened using the same protocol.

For each positive sample identified by real-time RT-PCR, nested RT-PCR targeting two coding regions of the enterovirus genome, VP1 and VP2, were performed (for primer details, see S1 Table), as described previously [4, 35–37]. PCR products were visualized on a 2% agarose gel stained with GelRed (Biotium). Each positive amplicon was then sequenced in both directions (SEQLAB GmbH, Germany).

#### Phylogenetic analysis of NHP enteroviruses

The VP1 and VP2 sequences obtained were compared to a dataset of complete sequences of all serotypes available in GenBank in order to determine whether these sequences were genetically related to any known EV serotypes. Then multiple alignments of all sequences were done using the ClustalW algorithm implemented in the MEGA 5 software package [38]. Phylogenetic trees were constructed by Maximum likelihood (ML) algorithm. The best-fitting ML model as determined using MODELTEST was GTR (General Time Reversible) [39]. The highest-likelihood DNA tree and the corresponding bootstrap support values were obtained by PhyML (freely available at the ATGC bioinformatics platform [40, 41]), using NNI (Nearest Neighbor Interchange)+SPR (Sub-tree Pruning Regrafting) branch swapping and 100 bootstrap replicates [42].

#### NHP species and subspecies characterization

It has been shown that the provisional species determination of NHPs in the field matched the results obtained with mitochondrial DNA sequencing for 97.3% of samples [27]. However, the host species had to be confirmed for all samples under study. Thus, DNA was extracted from fecal samples using QIAamp Stool DNA Mini kit (Qiagen, Valencia, CA) according to manufacturer’s recommendations. A mitochondrial DNA sequence of 386 bp spanning the 12S gene (primers 12S-L1091 5’-AAAAAGCTTCAAACTGGGATTAGATACCCCACTAT -3’ and 12S-H1478 5’-TGACTGCAGAGGGTGACGGGCGGTGTGT-3’) was amplified by PCR [43]. After visualization on a 2% agarose gel stained with GelRed (Biotium), each positive amplicon was sequenced in both directions (SEQLAB GmbH, Germany). Three assays were performed on each sample. The host species was confirmed by Basic Local Alignment Search Tool (BLAST, http://blast.ncbi.nlm.nih.gov/Blast.cgi).

#### Identification of each NHP individual by microsatellite analysis

In order to exclude fecal samples that could have been collected from the same host individual, microsatellite and gender analysis were performed on all EVs positive samples. Samples were genotyped at ten different previously described microsatellite loci (D2s1326, D3s1768, D5s1457, D6s1280, D7s817, D8s1106, D9s910, D13s765, D16s265, D18s536, see S2 Table) [44]. For the gender determination, the amelogenin gene (which contains a deletion in the X chromosome but not in the Y) was amplified using the primers AmelA (label5’-CCCTGGGCTCTGTAAAGAATAGTG-3’) and AmelB (5’-ATCAGAGCTTAAACTGGGAAGCTG-3’) [45]. PCR reactions were performed in a final volume of 10 μl (per sample per locus) of a mixture containing 5 μl of a 2X Qiagen multiplex PCR Master Mix, 0.5 μl of each primers at 2 μM, 1 μl of Qsolution 5X, 2.5 μl of water and 1 μl of DNA. Thermal conditions used to amplify all loci were 10 min at 95°C, followed by 45 cycles of 30 s at 94°C, 90 s at 58°C, 90 s at 72°C, and a final extension step of 10 min at 72°C [46]. Amplicons were analyzed using 3130xl Genetic Analyzer (Applied Biosystems, Foster, CA). Alleles were visualized and sized using Genemapper v4.0 software (Applied Biosystems). Homozygous samples were amplified a minimum of six times in order to exclude allelic dropout [27]. Samples that did not show successful results after six PCRs assays and two independent DNA extractions, as well as those presenting incomplete or multiple allelic profiles were discarded from the analysis.

### Enterovirus full-length genome sequencing

Enterovirus full-length genome sequencing was done for two positive samples detected in two different chimpanzees and one identified in a mandrill using two different strategies:

#### Genome walking strategy

Genome walking was performed for the three samples as described previously [47, 48]. To obtain the complete genome, partial region of the 5’-UTR, VP2, VP1 and 3D were amplified using previously published assays [35–37]. Following nucleotide sequencing of all PCR products, specific primers were designed for each virus. cDNA was generated using the SuperScript III kit (Life Technologies) and long range PCR-based amplification of 4 kb fragments was done using the Expand High Fidelity Plus kit (Roche). These PCR products were sequenced directly on both strands by primer walking. The 3’ terminus was amplified using the 5’/3’ RACE kit, 2^nd^ Generation (Roche).

#### High throughput sequencing

This strategy was used if the complete genome was not obtained by genome walking despite repeated attempts. RNA was reextracted with QIAmp Viral RNA Mini kit (Qiagen) according to the manufacturer’s instruction and treated with Turbo DNA-free kit (Ambion) to remove contaminating DNA. RNA was reverse transcribed into cDNA with SuperScript III reverse transcriptase using random hexamer primers (Life Technologies). Generated cDNA was then amplified with Phi29 enzyme provide in QuantiTect Whole Transcriptome kit (Qiagen), as described previously [49]. A barcoded library was prepared using the Nextera XT kit (Illumina). Amplified DNA was fragmented into 350 bp and adapters were added for multiplexing samples within the same channel. Sequencing was performed with Illumina Hi-seq 2000 sequencer (Illumina, San Diego, USA) as recommended by the manufacturer. All reads were filtered by quality (Phred score >20). Each viral read was selected using EVs complete genomes database and only the region that matched viral genome was considered. All reads were initially assembled by contigs with ABYSS software [50] with different values of *k*, and contigs were then assembled into a ‘super assembly’ with the CAP3 program [51, 52] in order to obtain the full-length viral genome.

### Recombination detection

Similarity plot and bootscanning analysis was done using SimPlot 3.5 [53]. Datasets for the analysis were selected to include potential recombination partners of sequenced viruses, but to exclude duplicate non-recombinant viruses and distantly related viruses not relevant for the analysis. Recombination analysis was also done with RDP 4.0 package [54]. To analyze ancient events in presence of significant phylogenetic noise, window size was increased to 300 nt for RDP, 400 nt for bootscan, 150 nt for MaxChi, 150 nt for Chimaera, 500 nt for SiScan. Other analysis parameters were set by default. To highlight interspecies recombination, the dataset included only four sequences: CVB3 strain Nancy (“classical” *EV-B*), EV103 (*EV-J*), EV68 *(EV-D*, an outgroup) and the query sequence, GAB130 or GAB653.

### Nucleotides sequences accession numbers

All sequences obtained in this study are available in GenBank under accession numbers KJ418235-KJ418243 for VP1, KJ418216-KJ418234 for VP2 and full length genomes under the following accession numbers KJ418244, KJ701248 and KJ701249 (see S4 Table).

## Results

### Non-invasive sampling of wild-living primates and EV molecular detection

The molecular identification of the host species, based on amplification of the 12S gene by PCR [43], was effective for 413 (68.83%) fecal samples out of the 600 collected. Specifically, 201 samples were identified as being collected from gorillas, 103 from chimpanzees, 76 from mandrills, 29 from black colobus, 3 from greater spot-nosed monkey and one from moustached monkey. The host species of 187 samples has not been identified after three assays, due to limited or degraded DNA.

Among the 413 high quality fecal samples, a total of 32 samples have been positive for EVs by real-time RT-PCR with Ct values between 30.09 and 41.07. Specifically, three positive samples were from La Lope, 23 from Ivindo, two from Mikongo and four from Lekedi (Fig 1). Among these 32 EV positive samples, 25 were identified and confirmed as being collected from mandrills and seven from chimpanzees. No EVs were detected in gorillas or in the three other monkey species studied. Microsatellite analysis revealed that the seven EV-positive chimpanzee samples corresponded to six distinct individuals, and that the 25 EV-positive mandrill samples represented 20 different individuals (S3 Table). Despite repeated attempts, three mandrill samples were not amplified, which could be due to either the nucleic acids degradation or the DNA availability (S3 Table). Thus, 26 different EVs have been identified in 20 mandrills and in 6 chimpanzees.

### Genetic diversity and phylogenetic relationships of NHP EVs

To genetically characterize EVs circulating in NHPs in Gabon, partial VP1 and VP2 sequences were amplified by nested RT-PCR. Despite repeated attempts (3 to 5 times), sequences of each coding region could not be obtained for all samples. Of the 26 samples, ten VP1 sequences were obtained (two from chimpanzees and eight from mandrills) and 21 VP2 sequences (four from chimpanzees and 17 from mandrills). This could be due to the fact that the PCR assays were originally designed to amplify human enteroviruses and could not take into account the sequence of simian viruses or poor RNA quality.

**Fig 2:**
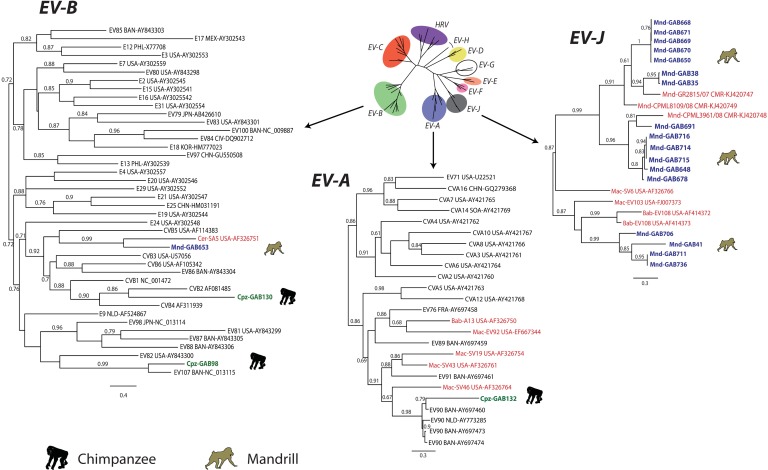
Phylogenetic relationship of EV strains based on approximately 450 nucleotides VP2 sequences (positions 962 to 1,545 according to the poliovirus 1 genome, Genbank # V01149), in the species *EV-B*, *EV-A* and *EV-J*. The genus tree was constructed using the partial VP2 sequences of representative serotypes of the genus *Enterovirus* by maximum likelihood method. Sequences obtained in this study are indicated in blue for mandrills and green for chimpanzees. Reference EV sequences of other NHP species are indicated in red. Names of NHP species are indicated (Mac for macaque, Bab for baboon, Cer for cercopothecus), countries are also indicated (BAN, Bangladesh; CHN for China; CIV for Ivory Coast; CMR for Cameroon; FRA for France; JPN for Japan; KOR for South Korea; MEX for Mexico; NLD for Netherland; PHL for Philippines; PUR for Puerto Rico; SLN for Slovenia; USA for United States of America; and SOA for Republic of South Africa). Trees were built using Maximum likelihood algorithm and GTR substitution model. Only bootstrap values ≥ 60% are indicated at the nodes. Scale bars represent the genetic distance. GenBank accession numbers of the sequences used are indicated in the tree (for details, see S4 Table).

**Fig 3:**
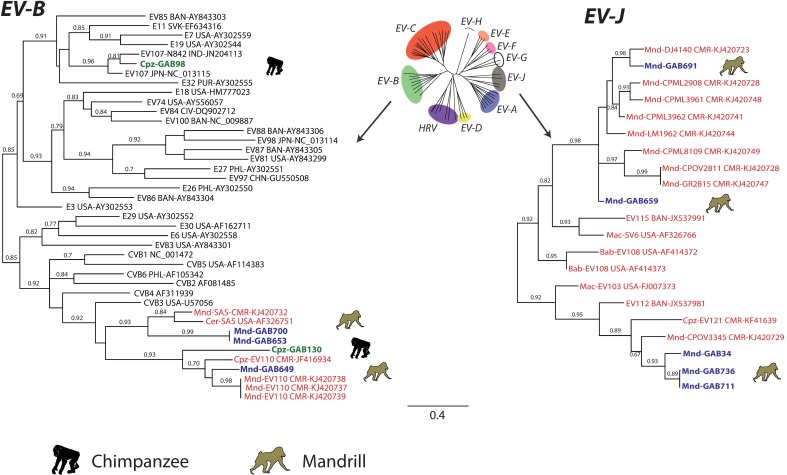
Phylogenetic relationship of EV strains based on approximately 320 nucleotides of the VP1 gene (positions 2,602 to 2,951 according to the poliovirus 1 genome, Genbank # V01149). Methods and designations are identical to Fig 2.

For the purpose of virus identification, we performed phylogenetic analysis of the mandrill- and chimpanzee-derived EVs using partial VP2 and partial VP1sequences (Figs 2 and 3). One chimpanzee EV VP2 sequence was too short and thus has not been included in phylogenetic analysis (GAB642). Phylogenetic trees based on VP2 and VP1 showed that all mandrill-derived EVs belonged to two EV species: *EV-B* (three sequences) and *EV-J* (18 sequences) (Figs 2 and 3).

Enterovirus type assignment is based on 75% sequence identity cut-off in the full VP1 genome region (ca. 900 nt) [55, 56]. There are no strict type criteria for the partial VP2 and VP1 genome regions that could be identified for most viruses in this study. Identification of a full VP1 sequence was attempted, but was not successful for most samples because of low quantity of viral RNA as evidenced by high threshold cycles in real-time PCR (data not shown). Therefore, a surrogate stringent cut-off of 80% sequence identity was used to identify known EV types. Within the *EV-J*, mandrill-derived EVs clustered with simian variants already identified in mandrills from Cameroon [19]. According to the proposed type assignment of the species *EV-J* [19], only one virus (GAB691) grouped with one of the five proposed types, the type 3 DJ4140/09 (Fig 3). The VP1 sequences showed 83.1% nucleotide identity with DJ4140/09. Other EVs variants obtained in our study (GAB41, GAB706, GAB711 and GAB736) grouped with the simian EVs characterized in other OWM species such as EV103 (*Macaca mulatta*) and EV108 (*Papio sp*.). Nucleotide sequence identity suggested that they could represent two novel EV-J types one represented by sample GAB659 and another one by samples GAB34, GAB711 and GAB736. Based on the VP1 sequences analysis, the other mandrill-derived EVs fell into the species *EV-B*. One sequence (GAB649) clustered with EV110, a virus previously identified in a chimpanzee and a mandrill [8, 19]. Since the VP1 sequences obtained showed 83.6% to 84.8% nucleotide identity with these EV110 isolates, GAB649 could be assigned to EV110. Two mandrill-derived viruses amplified in our study (GAB653 and GAB700) clustered with the SA5 isolate (Fig 3), a vervet monkey-derived EV [12]. As a full genome sequence was identified for sample GAB653, it was possible to precisely identify its type. The VP1 identity of GAB653 and the most closely related SA5 was below the nucleotide sequence identity threshold of 75% (only 74% nucleotide identity with SA5). Because this value was lower than the type threshold, the sample GAB653 was proposed to the *Picornaviridae* Study Group (PSG) of the International Committee for the Taxonomy of Viruses (ICTV), which assigned GAB653 as a novel *EV-B* type named EV-B113 (http://www.picornastudygroup.com/types/enterovirus/ev_b_types.htm).

Besides mandrills, EVs were also identified in chimpanzees. The phylogenetic analyzes based on VP1 genome region showed that chimpanzee-derived EVs fell into the species *EV-B* (two samples, GAB98 and GAB130) (Fig 3). Within the species *EV-B*, GAB98 clustered with EV107 identified in humans in Asia, first in Japan and then in India [57, 58]. The VP1 sequence of GAB98 had 87% to 88.3% nucleotide identity with these EV107 isolates, and was assigned to type EV107. The other chimpanzee EV (GAB130) grouped with simian EV110 (Fig 2). The nucleotide identity of EV110 and GAB130 was 70.1 to 74.8%. Therefore, GAB130 was proposed to the PSG (http://www.picornastudygroup.com/types/enterovirus/ev_b_types.htm) and designated EV-B112.

Even though classification of EVs by the VP1 gene is the gold standard, the VP2 sequence has also been suggested for classification [37]. Based on the VP2 sequences, the mandrill species *EV-J* isolates could represent 6 novel types. The two chimpanzee viruses were confirmed as member of *EV-B* and one chimpanzee sequence fell within *EV-A*. In the *EV-A*, this virus (GAB132) clustered with a serotype identified in human, EV90 (Fig 2). This chimpanzee EV could be putatively assigned as EV90 however without a full VP1, sequence type assignment for viruses sequenced only in VP2 remains uncertain.

### Recombination analysis of two chimpanzee- and one mandrill-derived EV genomes

**Fig 4:**
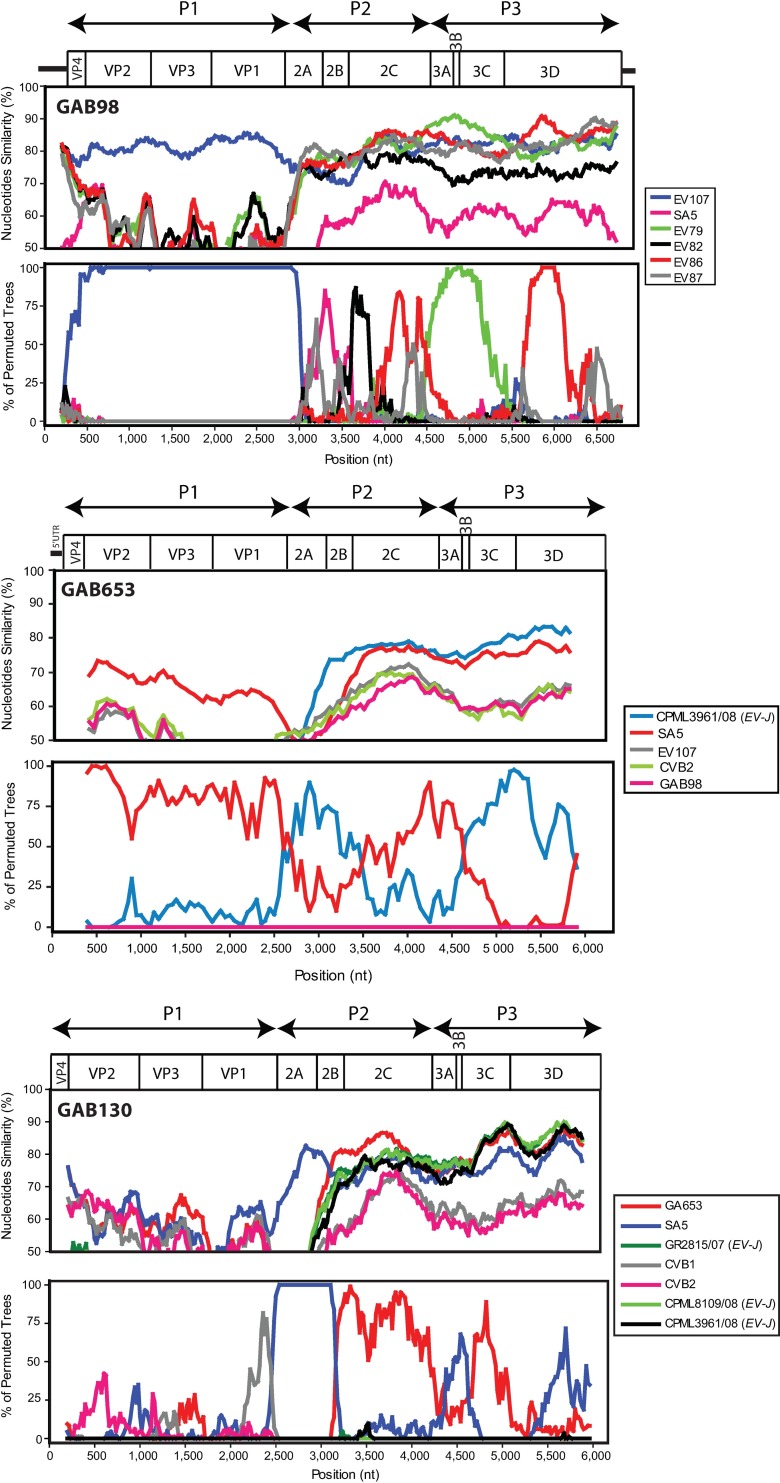
Similarity plot and bootscanning analysis of samples GAB98, GAB130 and GAB653. Similarity plot was performed using a sliding window of 400 nt moving with 20-nt step (800 nt window with 50-nt step for GAB653) and bootscanning was done using the Neighbor-joining method. The genome structure is indicated above the plot.

Full genomes for GAB98 and GAB653 were obtained by genome walking strategy, while for GAB130 by high throughput sequencing. For GAB130 a total of 5,674,506 RNA reads were obtained and the complete genome was constructed from 1261 reads. Similarity plot and bootscan analysis was performed on the complete coding regions with the sequences that were the most closely related to viruses sequenced here in the VP1, 2C and 3D genome regions. Strain GAB98 was most similar to EV107 in the complete capsid-encoding region P1. In the non-structural genome region, strain GAB98 was mosaic with regions of high identity to a number of *EV-B* strains (Fig 4).

**Fig 5:**
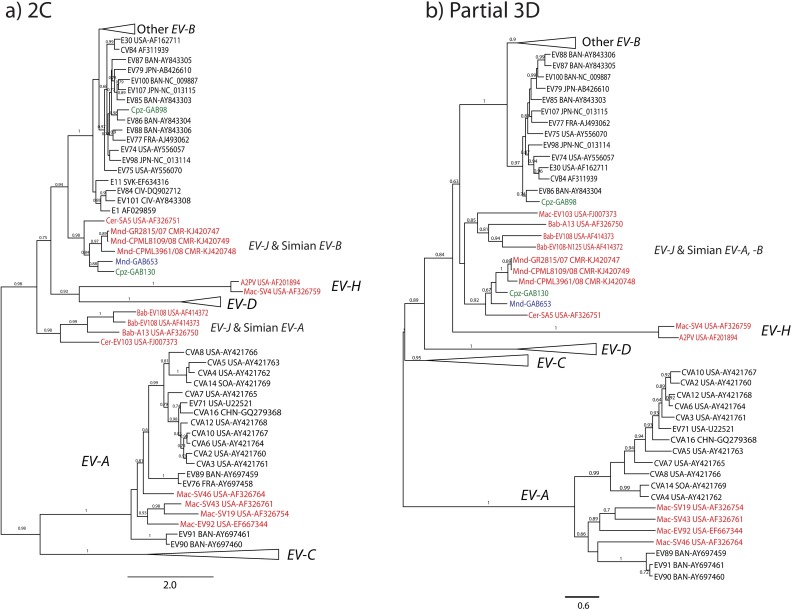
Phylogenetic relationship of human and NHP *EV-B* based on the complete 2C gene (a) and 935 nucleotides of the 3D region, from position 5,979 to 6,914 of the PV1 Mahoney strain (GenBank #V01149) (b). Designations are identical to Fig 2.

Since the whole genome of EV110 was not available, the prototype SA5, which was the next closest serotype on the phylogenetic trees, was used for the similarity plot and bootscan analysis of GAB130 and GAB653. In the P1 genome region, these viruses were on average less than 75% identical to SA5 and other viruses, concordant with their designation as novel types. In the non-structural genome region, there was evidence of interspecies recombination events. Species *EV-B* isolates GAB130 and GAB653 apparently acquired *EV-J* like non-structural proteins (Fig 4). The same kind of recombination event was also apparent in the previously published sequences of strains SA5, CPML8109/08, CPML3961/08, GR2815/07. It is likely that these phylogenetic conflicts reflect a single inter-species recombination event involving *EV-B* capsid genes and *EV-J* non-structural genes that produced this group of enteroviruses. Further mosaic recombination events are evident within this group in the non-structural genome region (Fig 4). Interspecies recombination observed on phylogenetic trees was confirmed using RDP package [54]. Phylogenetic conflict among CVB3 strain Nancy (*EV-B* species), Enterovirus 103 strain POo-1 (*EV-J* species) and isolate GAB130 with a breakpoint located approximately at genome position 4500 (middle of 2C protein) was identified by RDP, Bootscan, MaxChi, Chimaera, SiScan algorithms with P-values between 1.4x10^-15^ and 3.2x10^-5^. This recombination event was consistent with the conflict between phylogenetic trees for VP1 and 3D genome regions (Figs 3 and 5B). Location of the breakpoint within 2C region explains why relations of “classical” *EV-B*, *EV-J* and the SA5-like *EV-B* group were poorly resolved on the 2C genome region tree (Fig 5A).

According to the phylogenies based on partial VP1 sequences, GAB98, GAB130 and GAB653 samples belong to the species *EV-B*. Phylogenies of the non-structural regions 2C and 3D were compared to the VP1 based phylogeny. Based on 2C and 3D, the GAB98 isolate did not cluster with EV107 and was closer to EV86 (Fig 5A and 5B). Moreover, based on 3D, GAB130 and GAB653 (as well as the whole SA-5 like group of simian EVs) grouped with mandrill-derived *EV-J* isolates from Cameroon (Fig 5B). Therefore, phylogenetic analysis in 2C and 3D genome regions confirmed intraspecies recombination events suggested by bootscan analysis and provided evidence of an interspecies recombination event in simian EVs that involved *EV-B* and *EV-J* species.

## Discussion

The recent discovery of human EVs in fecal samples of wild NHPs in Cameroon [8] highlights the risk of transmission of EVs between NHPs and humans. Indeed, authors showed high rates of EV infection (95%) and suggested that was the consequence of the mandrills large population size, which could maintain viruses over long time periods through transmission between individuals [19]. The objective of the present study was to characterize the genetic diversity of EVs circulating in NHPs in Gabon and to identify the phylogenetic relationship of these EVs with human types. Our work showed co-circulation of human and simian EVs in NHPs in Gabon and allowed the characterization of two new *EV-B* types.

### Classification of mandrill EVs

We described the circulation of species *EV-B* and *EV-J* in wild mandrill populations of Gabon. Within the simian *EV-B* subgroup, GAB649 could be assigned to the type EV110, which was identified for the first time in a wild chimpanzee and then in mandrills in Cameroon [8, 19]. Considering that in Central Africa the distribution of mandrills and great apes are juxtaposed and that enteroviruses can be stable in the environment for a long period [59], this result suggests the interspecies transmission of this virus between mandrills and chimpanzees. The samples GAB653 and GAB700 grouped with other simian EVs, such as SA5 (isolated from *Cercopithecus aethiops*) [60]. According to the established type criteria, virus GAB653 was assigned to a novel type EV-B113.

Most of the mandrill-derived EVs detected in this study were members of the species *EV-J*, which is consistent with the previous study made on EVs of NHPs in Cameroon from OWM species including mandrills [19]. One of the five sequences obtained in this study clustered with members of the type 3. Other mandrill EV-J isolates probably constituted 6 novel types, but because of the lack of full VP1 sequences, they could not be reliably identified as novel or known types. Identification of EVs in NHPs is complicated because it relies on use of highly degenerate PCR primers that were originally designed to amplify only species *EVA*—*EV-D* [4, 35–37] and are poorly suitable for *EV-J*. The use of degenerated primers associated to the low quantity of viral RNA in samples complicated reliable identification of *EV-J* types.

Based on the observation that the EVs obtained in our study are divergent from those identified in Cameroon and likely represented multiple distinct types, it is likely that the diversity of EVs in mandrills in Central Africa remains underestimated. This has to be further investigated using specific primers for amplification of the complete VP1 of *EV-J*.

### Chimpanzee EVs classification

One sample, GAB132, belonged to the human type EV90. Of notice, EV90 was isolated from stool specimens of humans presenting with acute flaccid paralysis (AFP) or from healthy subjects in Asia and Europe during AFP surveillance [56, 61, 62]. Phylogenetic data also showed that two chimpanzee-derived EVs (GAB98 and GAB130) belong to another human EV species, *EV-B* (Figs 2 and 3). Indeed, the sample GAB98 belonged to type EV107, which has been isolated from a Japanese traveler returning from Thailand and Nepal [57]. Sample GAB130 clustered within the species *EV-B* and was identified as a novel type, EV-B112.

### Implications for enterovirus taxonomy

Designation of the formerly “human” enterovirus species A-D was easy because they were genetically distinct over the whole coding region, and clear sequence distance criteria of species could be used. Human enteroviruses commonly recombine within a species, but never between species [63]. Recombination in circulating human EVs occurs every few years [64, 65] and may have been a genetic force that shaped the relatively genetically uniform species *EV-A–EV-D*. The discovery of a group of simian species *EV-B* strains that have distinct EV-J like non-structural proteins here and in previous studies [19, 66] challenges the current taxonomic criteria. Indeed, these viruses are indistinguishable from the classical EV-B types in VP1 (Fig 2), but in 2C and 3D genome regions form a distinct cluster that is more similar to the species *EV-J* (Fig 5). These simian EV-B strains have evidence of additional mosaic recombination in the non-structural genome region, just as other EVs, but only within their group, not with classical EV-B types and not with the distinct EV-J types (EV103 and EV108). Noteworthy, by a combination of genetic properties these simian EV-B strains differ from typical EV-B types more than the two major clusters of EV-A species, termed A1 and A2, differ from each other [56]. As suggested earlier [19], this challenge needs to be considered by the ICTV study group.

### Cross-species transmission

Detection of species *EV-A* and *EV-B* in chimpanzees implies that they may be infected with these viruses, consistent with previously published observations [67]. Circulation of the same virus (EV107) in human populations in Asia and the detection of this virus in African wild primate living in a forest with minimal human contacts implies relatively easy long distance transmission of the virus, probably via humans. This opportunity have been enhanced recently because the Gabonese Government signed agreements with Asian companies involved in roads constructions and forestry. These companies have built quarters located at or around natural forests for Asian and Gabonese workers, increasing contacts between humans and wild animals. Such cross-species not only constitute a potential threat for humans, but can also result in morbidity in two endangered species, in chimpanzees [11], and probably in mandrills, too. The discovery of EV110 in both a chimpanzee and a mandrill is consistent with a previous study made in Cameroon [19] and confirms the role of chimpanzees and mandrills in circulation of EVs.

The evolutionary origin of the EV-B group with EV-J non-structural proteins remains unclear. It could be a result of a recent cross-species transmission and inter-species recombination. Another possibility could be the co-evolution of EVs with primates. Enterovirus B types that are commonly found in humans have been detected in NHPs in our study (e.g. Cpz-GAB130 and Cpz-GAB98) and in previous reports [67]. However, there have been no data on detection of the EV-B/EV-J recombinant viruses in humans. Moreover, in VP1 these viruses lay aside from the "classical" human EV-B (Fig 3). So far, it presents that this group of EV-B is specific to primates, and may represent current emergence of a novel species from EV-B. Unfortunately, it is not possible to definitively conclude if this group emerged recently or co-evolved with primates for some time because in EVs mutations reach saturation on a century scale [68], and analysis of more ancient events would be doubtful.

### Genetic diversity of EVs

Here, we showed the circulation of two new types of EVs of the species *EV-B*, one in a chimpanzee and one in a mandrill, in Gabon. The discovery of two new types in a relatively small sample highlights that the diversity of EVs is largely underestimated. It may be speculated that the diversity of EVs in diverse and often ecologically separated NHPs would greatly exceed that known today. Further research of NHP EVs would help understanding emergence of new types and virus variants, including highly pathogenic ones, and evaluating the real risk of cross-species transmission for humans as well as for NHP populations.

## Supporting Information

S1 Fig Coverage of complete genome assembly from GAB98 (A), GAB130 (B) and GAB653 (C). GAB98 and GAB653 genomes were obtained by genome walking strategy while GAB130 was obtained by high throughput sequencing method. Enterovirus genome organization had been shown and the red bold lines mean the part of the genome successfully obtained.
(EPS)

S1 Table Primers and probes used to amplify enteroviruses by real-time RT-PCR or PCR. Location of each primer/probe is given in relation to the reference genome PV1 strain Mahoney (Genbank accession number V01149).
(DOCX)

S2 Table Microsatellite primers used to determine the number of individual positive samples. Locus name, primer sequence and expected size are indicated.
(DOCX)

S3 Table Genotypes obtained by microsatellite analysis, based on 10 markers, for the 32 EVs positive NHP samples obtained in this study. For each location, the zone code is indicated as following: IV for Ivindo, LE for Lékédi, LO for Lopé, and MI for Mikongo. F for female and M for male. NA corresponds to the values not available.
(DOCX)

S4 Table GenBank accession numbers of sequences obtained in this study. (DOCX)
