# Supplementary material for: African Non-Human Primates Host Diverse Enteroviruses
Source: PLoS One. 2017 Jan 12;12(1):e0169067. doi: 10.1371/journal.pone.0169067 (PMC5233426; doi:10.1371/journal.pone.0169067)
Supplement: S1 Table — Location of each primer/probe is given in relation to the reference genome PV1 strain Mahoney (Genbank accession number V01149). (DOCX) [file pone.0169067.s002.docx]

| Primer name | Sequence (5’-3’) | Genome region targeted | Genome location | References |
| --- | --- | --- | --- | --- |
| EQ-1 | ACATGGTGTGAAGAGTCTATTGAGCT | 5’-UTR | 437-462 | [1] |
| EQ-2 | CCAAAGTAGTCGGTTCCGC |  | 560-578 |  |
| EP-mod | FAM-ATTAGCCGCATTCAGGGGCCGGA-BHQ1 |  |  | Drexler *et al*, unpublished |
| 224 | GCIATGYTIGGIACICAYRT | VP3 | 1977-1996 | [2] |
| 222 | CICCIGGIGGIAYRWACAT | VP1 | 2969-2951 | [3] |
| AN89 | CCAGCACTGACAGCAGYNGARAYNGG |  | 2602-2627 | [2] |
| AN88 | TACTGGACCACCTGGNGGNAYRWACAT |  | 2977-2951 |  |
| AM11 | GARGCITGYGGITAYAGYGA | VP2 | 962-981 | [4] |
| AM12 | GARGARTGYGGITAYAGYGA |  | 962-981 |  |
| AM21 | GGITGGTGGTGGAARYTICC |  | 1178-1197 |  |
| AM22 | GGITGGTAYTGGAARTTICC |  | 1178-1197 |  |
| AM31 | TTDATDATYTGRTGIGG |  | 1545-1529 |  |
| AM32 | TTDATCCAYTGRTGIGG |  | 1545-1529 |  |
| EV_6590F | GCIGTIGGITGYRAYCCIGA | 3D |  | (Drexler *et al*, 2010 unpublished) |
| EV_6820F | ATGCCITCIGGITGYTCWGGIAC |  |  |  |
| EV_7000R | YTTRTCWGSWGGWGTCAT |  |  |  |

**S1 Table**. Primers and probes used to amplify enteroviruses by real-time RT-PCR or PCR. Location of each primer/probe is given in relation to the reference genome PV1 strain Mahoney (Genbank accession number V01149).

**References**

1. Dierssen U, Rehren F, Henke-Gendo C, Harste G, Heim A. Rapid routine detection of enterovirus RNA in cerebrospinal fluid by a one-step real-time RT-PCR assay. Journal of clinical virology : the official publication of the Pan American Society for Clinical Virology. 2008;42(1):58-64. doi: 10.1016/j.jcv.2007.11.016. PubMed PMID: 18164234.

2. Nix WA, Oberste MS, Pallansch MA. Sensitive, seminested PCR amplification of VP1 sequences for direct identification of all enterovirus serotypes from original clinical specimens. J Clin Microbiol. 2006;44(8):2698-704. doi: 10.1128/JCM.00542-06. PubMed PMID: 16891480; PubMed Central PMCID: PMC1594621.

3. Oberste MS, Maher K, Flemister MR, Marchetti G, Kilpatrick DR, Pallansch MA. Comparison of classic and molecular approaches for the identification of untypeable enteroviruses. J Clin Microbiol. 2000;38(3):1170-4. PubMed PMID: 10699015; PubMed Central PMCID: PMC86366.

4. Nasri D, Bouslama L, Omar S, Saoudin H, Bourlet T, Aouni M, et al. Typing of human enterovirus by partial sequencing of VP2. J Clin Microbiol. 2007;45(8):2370-9. doi: 10.1128/JCM.00093-07. PubMed PMID: 17537940; PubMed Central PMCID: PMC1951248.
