# Supplementary material for: African Non-Human Primates Host Diverse Enteroviruses
Source: PLoS One. 2017 Jan 12;12(1):e0169067. doi: 10.1371/journal.pone.0169067 (PMC5233426; doi:10.1371/journal.pone.0169067)
Supplement: S2 Table — Locus name, primer sequence and expected size are indicated. (DOCX) [file pone.0169067.s003.docx]

**S2 Table**. Microsatellite primers used to determine the number of individual positive samples. Locus name, primer sequence and expected size are indicated.

| Locus | Sequence 5’-3’ | | Amplicon size |
| --- | --- | --- | --- |
|  | Forward | Reverse |  |
| D9s910 | AAGTCAGTTAGCTGAAGGTTGC | TATATGAAGTGCTTAGAAAAAGTGC | 101/122 bp |
| D5s1457 | TAGGTTCTGGGCATGTCTGT | TGCTTGGCACACTTCAGG | 128 bp |
| D8s1106 | TTGTTTACCCCTGCATCACT | TTCTCAGAATTGCTCATAGTGC | 158 bp |
| D3S1768 | GGTTGCTGCCAAAGATTAGA | CACTGTGATTTGCTGTTGGA | 205/217 bp |
| D6S128 | CTGAATTTAGTCAGGGGTTCC | TCCATCACATGAGCAATTTC | 201 bp |
| D13S765 | TGTAACTTACTTCAAATGGCTCA | TTGAAACTTACAGACAGCTTGC | 224 bp |
| D16S265 | CCAGACATGGCAGTCTCTA | AGTCCTCTGTGCACTTTGT | 88 bp |
| D18S536 | ATTATCACTGGTGTTAGTCCTCTG | CACAGTTGTGTGAGCCAGTC | 161 bp |
| D7s817 | TTGGGACCTCTTATTTTCCA | GGGTTCTGCAGAGAAACAGA | 164/200 bp |
| D2s1326 | GAACTCAGGCAGCTTTCTCA | TAATTGTGTGAGCCAGTTTCC | 259 bp |
