# Supplementary material for: African Non-Human Primates Host Diverse Enteroviruses
Source: PLoS One. 2017 Jan 12;12(1):e0169067. doi: 10.1371/journal.pone.0169067 (PMC5233426; doi:10.1371/journal.pone.0169067)
Supplement: S3 Table — For each location, the zone code is indicated as following: IV for Ivindo, LE for Lékédi, LO for Lopé, and MI for Mikongo. F for female and M for male. NA corresponds to the values not available. (DOCX) [file pone.0169067.s004.docx]

**S3 Table**. Genotypes obtained by microsatellite analysis, based on 10 markers, for the 32 EVs positive NHP samples obtained in this study. For each location, the zone code is indicated as following: IV for Ivindo, LE for Lékédi, LO for Lopé, and MI for Mikongo. F for female and M for male. NA corresponds to the values not available.

| Sample name | Host common name | Sex | Location | Microsatellites loci genotypes | | | | | | | | | |
| --- | --- | --- | --- | --- | --- | --- | --- | --- | --- | --- | --- | --- | --- |
|  |  |  |  | D3s1768 | D5s1457 | D9s910 | D8s1106 | D6s1280 | D13s765 | D7s817 | D2s1326 | D18s536 | D16s265 |
| GAB98 | Chimpanzee | F | LO | 168/176 | 104/124 | 119/128 | 156/156 | 204/224 | 193/201 | 176/180 | 243/255 | 138/148 | 98/98 |
| GAB99 | Chimpanzee | NA | LO | 168/176 | 104/124 | 119/128 | 156/156 | 204/224 | 193/201 | 176/180 | 243/255 | 138/148 | 98/98 |
| GAB100 | Chimpanzee | M | LO | 168/172 | 108/124 | 119/119 | 140/152 | 212/216 | 201/201 | 172/176 | 247/247 | 148/148 | 88/88 |
| GAB130 | Chimpanzee | F | MI | 172/172 | 112/112 | NA | NA | 172/172 | 193/193 | 172/172 | NA | 170/170 | 96/106 |
| GAB132 | Chimpanzee | NA | MI | 168/168 | 112/112 | 113/113 | 136/136 | 172/204 | NA | 172/176 | 243/243 | 176/176 | 88/88 |
| GAB642 | Chimpanzee | NA | IV | 168/172 | NA | 122/122 | NA | 204/204 | NA | NA | NA | NA | NA |
| GAB720 | Chimpanzee | F | IV | 172/172 | 120/128 | 116/119 | 140/144 | 204/208 | 201/201 | 204/208 | 247/247 | 148/174 | 100/100 |
| GAB34 | Mandrill | F | LE | NA | 120/120 | 113/113 | NA | 190/190 | 213/213 | 176/176 | 243/243 | 146/146 | NA |
| GAB35 | Mandrill | F | LE | 188/212 | 124/124 | 113/113 | 144/156 | 186/186 | 209/209 | 156/176 | 243/243 | 150/150 | 84/84 |
| GAB41 | Mandrill | NA | LE | NA | NA | NA | NA | NA | NA | NA | NA | NA | NA |
| GAB38 | Mandrill | F | LE | 200/200 | 124/124 | 110/113 | 150/150 | NA | NA | 176/192 | 271/271 | 146/146 | NA |
| GAB648 | Mandrill | F | IV | 170/236 | NA | NA | 170/158 | NA | NA | NA | NA | NA | 84/84 |
| GAB649 | Mandrill | NA | IV | NA | NA | NA | NA | NA | NA | NA | NA | NA | NA |
| GAB650 | Mandrill | NA | IV | NA | NA | NA | NA | NA | NA | NA | NA | NA | NA |
| GAB653 | Mandrill | F | IV | NA | 148/148 | 101/101 | NA | NA | NA | 208/208 | NA | NA | NA |
| GAB659 | Mandrill | F | IV | 208/212 | 116/116 | NA | NA | NA | NA | NA | NA | NA | NA |
| GAB668 | Mandrill | NA | IV | 172/172 | 108/108 | NA | 148/148 | NA | NA | 164/164 | 219/219 | 162/162 | NA |
| GAB669 | Mandrill | NA | IV | 184/184 | 108/108 | NA | 140/140 | 184/184 | 185/185 | 160/160 | NA | 136/162 | NA |
| GAB670 | Mandrill | F | IV | NA | 112/112 | 113/113 | 140/140 | NA | NA | NA | 247/247 | NA | 114114 |
| GAB671 | Mandrill | NA | IV | NA | 098/098 | NA | 154/148 | 182/182 | 205/205 | NA | NA | 132/132 | 78/84 |
| GAB678 | Mandrill | M | IV | 226/226 | 130/130 | 116/116 | 154/148 | NA | NA | 172/172 | NA | 168/168 | 80/80 |
| GAB682 | Mandrill | F | IV | NA | 128/128 | 116/119 | NA | 190/190 | 197/201 | 172/172 | NA | 168/168 | NA |
| GAB691 | Mandrill | F | IV | 208/208 | NA | 116/116 | NA | 190/190 | 201/201 | NA | 219/219 | 162/162 | 78/78 |
| GAB698 | Mandrill | F | IV | 188/188 | 112/128 | 113/113 | 158/158 | 182/186 | NA | 160/160 | NA | 136/158 | 84/84 |
| GAB700 | Mandrill | F | IV | 184/214 | 132/132 | 113/116 | 144/144 | 190/194 | 201/201 | 160/176 | 247/247 | 132/132 | 78/84 |
| GAB706 | Mandrill | F | IV | 208/226 | 108/128 | 113/113 | 144/150 | 182/186 | 205/205 | 176/176 | NA | 162/172 | 78/78 |
| GAB711 | Mandrill | F | IV | 226/226 | 134/134 | 110/110 | 154/154 | NA | NA | NA | NA | NA | 88/114 |
| GAB714 | Mandrill | F | IV | 196/214 | 144/148 | 110/110 | 140/144 | 190/190 | 205/205 | 172/172 | NA | NA | 84/84 |
| GAB715 | Mandrill | F | IV | 196/214 | 128/128 | 107/110 | 128/128 | 190/190 | 201/201 | 168/168 | 227/247 | 168/168 | NA |
| GAB716 | Mandrill | NA | IV | 196/214 | 128/128 | 107/110 | 140/144 | 190/190 | 201/205 | 172/172 | 243/247 | 162/162 | 84/84 |
| GAB736 | Mandrill | M | IV | 184/184 | 128/128 | 110/119 | 128/128 | NA | 209/209 | 212/212 | NA | NA | NA |
| GAB859 | Mandrill | M | IV | 188/218 | 128/128 | 113/113 | 136/144 | 190/194 | 201/201 | 168/172 | 247/247 | 162/162 | NA |
