# Supplementary material for: African Non-Human Primates Host Diverse Enteroviruses
Source: PLoS One. 2017 Jan 12;12(1):e0169067. doi: 10.1371/journal.pone.0169067 (PMC5233426; doi:10.1371/journal.pone.0169067)
Supplement: S4 Table — (DOCX) [file pone.0169067.s005.docx]

**S4 Table.** GenBank accession numbers of sequences obtained in this study.

| Name of the sample | GenBank accession numbers | | |  |
| --- | --- | --- | --- | --- |
|  | 12S | VP1 | VP2 | Full genome |
| GAB 34 | KJ476231 | KJ418235 | - | - |
| GAB 35 | KJ476232 | - | KJ418216 | - |
| GAB 38 | KJ476233 | - | KJ418217 | - |
| GAB 41 | - | - | KJ418218 | - |
| GAB 98 | KJ476234 | KJ418242 | - | KJ701248 |
| GAB 130 | KJ476237 | - | - | KJ418244 |
| GAB 132 | KJ476238 | - | KJ418219 | - |
| GAB 648 | KJ476240 | - | KJ418220 | - |
| GAB 649 | - | KJ418236 | - | - |
| GAB 650 | - | - | KJ418222 | - |
| GAB 653 | KJ476241 | KJ418237 | KJ418221 | KJ701249 |
| GAB 659 | KJ476242 | KJ418238 | - | - |
| GAB 668 | KJ476243 | - | KJ418223 | - |
| GAB 669 | KJ476244 | - | KJ418224 | - |
| GAB 670 | KJ476245 | - | KJ418225 | - |
| GAB 671 | KJ476246 | - | KJ418226 | - |
| GAB 678 | KJ476247 | - | KJ418227 | - |
| GAB 691 | - | KJ418239 | KJ418228 | - |
| GAB 700 | KJ476249 | KJ418243 | - | - |
| GAB 706 | KJ476250 | - | KJ418229 | - |
| GAB 711 | KJ476251 | KJ418240 | KJ418230 | - |
| GAB 714 | KJ476252 | - | KJ418231 | - |
| GAB 715 | KJ476253 | - | KJ418232 | - |
| GAB 716 | KJ476254 | - | KJ418233 | - |
| GAB 736 | KJ476256 | KJ418241 | KJ418234 | - |
